# New Metabolic Influencer on Oxytocin Release: The Ghrelin

**DOI:** 10.3390/molecules24040735

**Published:** 2019-02-18

**Authors:** Renáta Szabó, Rudolf Ménesi, Andor H. Molnár, Zita Szalai, Lejla Daruka, Gábor Tóth, János Gardi, Márta Gálfi, Denise Börzsei, Krisztina Kupai, Anna Juhász, Marianna Radács, Ferenc A. László, Csaba Varga, Anikó Pósa

**Affiliations:** 1 Department of Physiology, Anatomy and Neuroscience, Faculty of Science and Informatics, University of Szeged, 6726 Szeged, Hungary; szaborenata88@gmail.com (R.S.); vazoaktiv@gmail.com (R.M.); szalzita@gmail.com (Z.S.); dar.lejla@gmail.com (L.D.); borzseidenise@gmail.com (D.B.); kupai@bio.u-szeged.hu (K.K.); 408.labor@gmail.com (F.A.L.); vacs@bio.u-szeged.hu (C.V.); 2 Department of Physiology, Anatomy and Neuroscience, Interdisciplinary Excellence Centre, University Of Szeged, Szeged, Hungary; 3 Institute of Physical Education and Sport Sciences, Gyula Juhász Faculty of Education, University of Szeged, 6725 Szeged, Hungary; andor.h.molnar@gmail.com; 4 Department of Medical Chemistry, Faculty of Medicine, University of Szeged, 6720 Szeged, Hungary; toth.gabor@med.u-szeged.hu; 5 First Department of Internal Medicine, Faculty of Medicine, University of Szeged, 6720 Szeged, Hungary; gardi.janos@med.u-szeged.hu; 6 Department of Environmental Biology and Education, Gyula Juhász Faculty of Education, Institute of Applied Science, University of Szeged, 6725 Szeged, Hungary; galfi@jgypk.u-szeged.hu (M.G.); radacs@jgypk.u-szeged.hu (M.R.); 7 Department of Psychiatry, Faculty of Medicine, University of Szeged, 6725 Szeged, Hungary; 8 HR-Pharma Ltd., 6726 Szeged, Hungary

**Keywords:** ghrelin, ghrelin antagonist, oxytocin, neuropeptide regulation

## Abstract

Background: The hypothalamic–pituitary axis by secreting neuropeptides plays a key role in metabolic homeostasis. In light of the metabolic regulation, oxytocin is a potential neuropeptide for therapies against obesity and related disorders. The aim of our study is to measure ghrelin-induced oxytocin secretion in rats and to detect the changes after administration of ghrelin antagonist. Methods: Ghrelin was administrated centrally (intracerebroventricular, i.c.v., 1.0, 10.0, and 100.0 pmol) or systemically (intravenous, i.v., 1.0, and 10.0 nmol). [d-Lys^3^]-GHRP-6 ghrelin antagonist was injected 15 min before ghrelin injection in a dose of 10.0 pmol i.c.v. and 10.0 nmol i.v. Results: Either i.c.v. or i.v. administration of ghrelin dose-dependently increased the plasma oxytocin concentration. Following pretreatment with the ghrelin antagonist [d-Lys^3^]-GHRP-6, the high plasma oxytocin level induced by ghrelin was significantly reduced. Conclusion: The results indicate that the release of oxytocin is influenced directly by the ghrelin system. Examination of the mechanism of ghrelin-induced oxytocin secretion is a new horizon for potential therapeutic options.

## 1. Introduction

The increasing prevalence of energy metabolism disorders and diseases are both a public health issue and clinical problem. It has been established that the energy homeostasis plays a role in controlling the synthesis and secretion of various hormones, thus any disturbance in these contributes to the impairment of the metabolic regulation. The central nervous system (CNS), especially the hypothalamus and pituitary, are important to promote the action of the metabolic regulatory mediators and influence, e.g., the appetite [1]. 

Ghrelin is a 28-amino acid-containing endogenous ligand for the growth hormone secretagogue receptor 1a (GHS-R1a) linking the CNS with peripheral tissues that regulate food intake and energy homeostasis. The expression of ghrelin has been demonstrated in various tissues, such as the stomach, kidney, pituitary gland and, hypothalamus [2]. Previous studies suggest that ghrelin could contribute to metabolic disturbances. Low plasma ghrelin is associated with insulin resistance, hypertension, and increase the risk of type 2 diabetes [3] and metabolic syndrome [4]. 

In our previous study, we reported that vasopressin and oxytocin secretion are increased following the intracerebroventricular (i.c.v.) or intravenous (i.v.) administration of ghrelin in cell cultures of neurohypophyseal tissue. The results indicate that vasopressin and oxytocin release are influenced directly by the ghrelin system, and the effects of ghrelin on vasopressin and oxytocin secretion from the neurohypophyseal tissue in rats can occur at the level of the posterior pituitary [5].

In light of the importance of hypothalamic–pituitary axis and its mediators in metabolic regulation, oxytocin has a pivotal role in the energy homeostasis. Activation of central and peripheral oxytocin receptors also regulates energy expenditure [6]. Oxytocin promotes glucose uptake and stimulates insulin secretion [7]. Zhang et al. found that oxytocin improved the lipid profile by lowering serum low density lipoprotein and cholesterol levels [8]. Summarizing the results it can be concluded that hypothalamic–pituitary pathways and its neuropeptides are closely linked to metabolic regulation. Hence, the examination and identification of the neuroendocrine processes are necessary to understand the exact effects of neuropeptides, which promote further studies for metabolic changes. 

In our previous work we proved that ghrelin administration enhances oxytocin secretion in neurohypophyseal cell cultures. Based on our in vitro results, the aim of our current study is to examine the effects of centrally and systemically administrated ghrelin on oxytocin release in rats. Furthermore, the effect of ghrelin receptor (GHS-R1a) antagonist [d-Lys^3^]-GHRP-6 has been also measured on plasma oxytocin secretion. 

## 2. Results

### 2.1. Effects of Centrally (i.c.v.) and Systemically (i.v.) Administrated Ghrelin on Oxytocin Secretion

**Figure 1:**
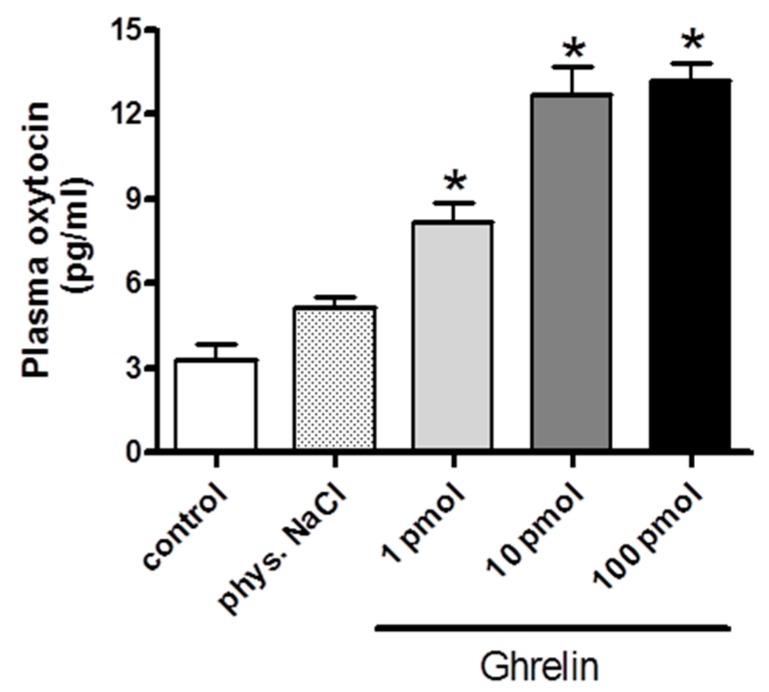
Dose–response effects of centrally (i.c.v.) administrated ghrelin on plasma oxytocin release (expressed as pg/ml). Results shown as means ± S.E.M., *n* = 10. Statistical significance: * *p* < 0.05 relative to the control group.

Following the i.c.v. administration of 1 pmol ghrelin, the plasma oxytocin level significantly increased. After administration of a higher ghrelin dose (10 pmol and 100 pmol), the oxytocin secretion was further enhanced, but no significant difference was observed between the effects of 10 or 100 pmol doses. Data are presented in Figure 1.

**Figure 2:**
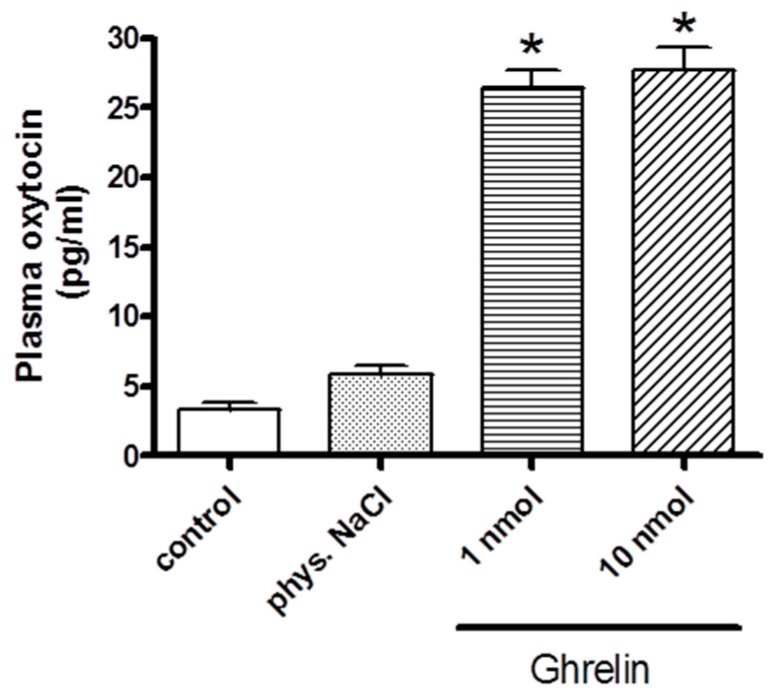
Effects of systemically (i.v.) administered ghrelin on oxytocin secretion (expressed as pg/ml). Results shown as means ± S.E.M., *n* = 10. Statistical significance: * *p* < 0.05 relative to the control group.

As shown in Figure 2, i.v. injections of ghrelin significantly enhanced the plasma oxytocin values. Both 1 and 10 nmol doses of ghrelin resulted same elevation in oxytocin level. 

### 2.2. Effects of Centrally (i.c.v.) and Systemically (i.v.) Administrated Ghrelin Antagonist on Oxytocin Secretion

**Figure 3:**
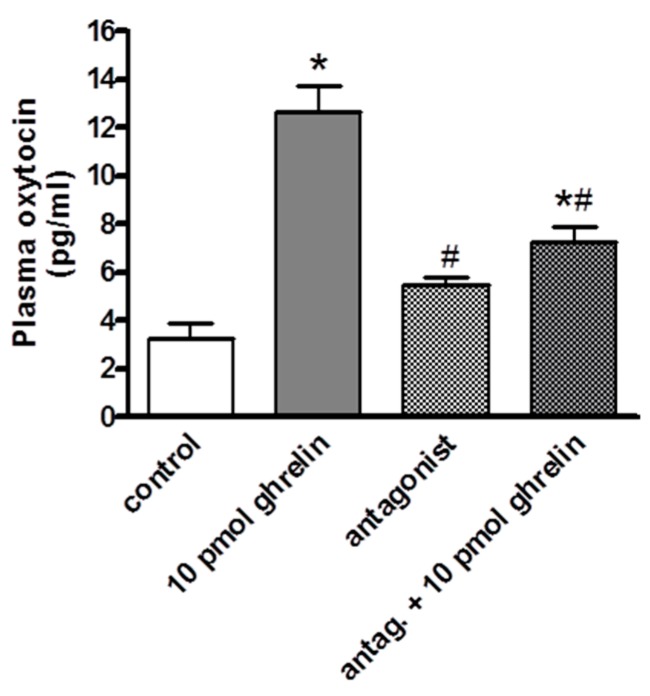
Effects of centrally (i.c.v.) administered ghrelin antagonist [D-Lys3]–GHRP-6 on the plasma oxytocin concentration (expressed as pg/mL). Results shown as means ± S.E.M., *n* = 10. Statistical significance: * *p* < 0.05 relative to the control group and # *p* < 0.05 relative to the 10 pmol ghrelin-treated group.

The i.c.v. administered ghrelin antagonist [d-Lys^3^]-GHRP-6 did not induce any significant changes in oxytocin concentration compared to the control group. However, the higher oxytocin levels induced by ghrelin were significantly decreased, though the plasma oxytocin concentration remained above the control level. Data are presented in Figure 3.

**Figure 4:**
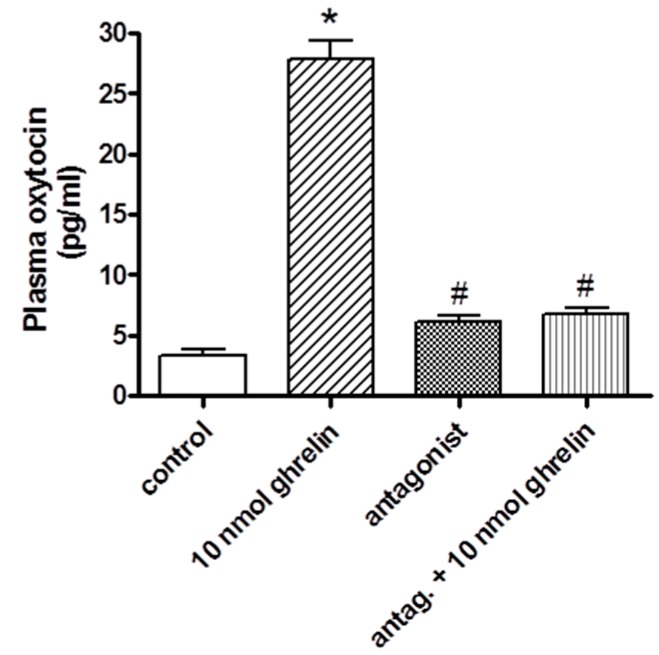
Effects of systemically (i.v.) administered ghrelin antagonist on the plasma oxytocin level (expressed as pg/mL). Results shown as means ± S.E.M., *n* = 10. Statistical significance: * *p* < 0.05 relative to the control group and # *p* < 0.05 relative to the 10 pmol ghrelin-treated group.

After the i.v. administration of the ghrelin antagonist, changes in the oxytocin concentration were not observed. The high plasma oxytocin levels induced by ghrelin were fully blocked, and the control level was observed. Data are presented in Figure 4.

### 2.3. Behavioral Changes at the End of the Experimental Period

Available data suggest that ghrelin possesses a dual role in stress and related behavioral disorders [9] as well as it can change feeding behavior [10]. During this 30-min-experiment, we did not observe behavioral changes or a significant change in the consumption of the rats.

## 3. Discussion

A growing body of evidence indicates that metabolic disorders are significantly linked to the dysregulation of the CNS. Considering the importance of hypothalamic–pituitary axis in metabolic regulation, the objective of our current study was to investigate the basic regulatory pathways at the site of hypothalamus/pituitary [11]. Numerous studies examine and prove the effects of oxytocin as a potential therapeutic option [12]. Using an animal model, Camerino reported that oxytocin or oxytocin receptor knockout mice gained weight combined with impaired glucose homeostasis [13]. Experiments in rodents and preclinical studies show that exogenous oxytocin administration reduces caloric consumption and produces weight loss [14,15,16]. We used an alternative way by administrating (i.c.v or i.v.) of ghrelin to increase oxytocin signaling. 

As regards the mechanism of ghrelin, we presume that ghrelin stimulates oxytocin release by acting directly on oxytocin-producing neurons in the hypothalamic paraventricular or supraoptic nuclei [17,18]. Previous data suggest that i.c.v. administration of ghrelin increases c-Fos immunoreactivity, which shows that ghrelin modulates neuronal activity in the hypothalamic paraventricular nucleus [19]. Consequently, there is a functional link between ghrelin and oxytocin producing cells/ oxytocin neuropeptide. Olszewski et al. demonstrated that oxytocin serves as a negative feedback regulator in feeding-related mechanisms driven by ghrelin. Interplay of ghrelin and oxytocin may limit ghrelin-induced excessive level of food intake, whereas interactions with orexigenic peptides (e.g., orexin and neuropeptide Y) promote ghrelin-induced food intake [10]. Based on our earlier observations related to neurohypohyseal oxytocin secretion, the stimulatory effect between oxytocin and ghrelin seems to be similar by i.c.v. and i.v. administration of ghrelin. The mode of the oxytocin-increasing action of systemically administered ghrelin has not been fully clarified. Circulating ghrelin cannot penetrate the blood–brain barrier and activate the hypothalamic magnocellular cells since the ghrelin molecule is too large to pass through the blood–brain barrier. It seems that oxytocin is released directly from the neurohypophysis. We earlier reported that dispersed cell cultures of neurohypophysis isolated from adult rats are capable of synthesizing and releasing oxytocin [20,21]. Our observations on neurohypophyseal cultures proved the significant role of pituicytes in the ghrelin-induced changes in oxytocin excretion. To verify that ghrelin influences oxytocin secretion, we carried out examinations with a specific ghrelin receptor antagonist. The synthetic ghrelin antagonist [d-Lys^3^]-GHRP-6 (His-DTrp-DLys-Trp-DPhe-Lys-NH_2_) is widely applied under various experimental conditions [22]. We observed that the higher plasma oxytocin level induced by ghrelin was significantly decreased by the ghrelin antagonist administered before the ghrelin injection. 

It is interesting that ghrelin antagonists could also moderate the neurohypophyseal hormone release-increasing effect of ghrelin only if the antagonists are administered before ghrelin treatment [5]; if ghrelin administration precedes application of the antagonists, the ghrelin receptor antagonists prove ineffective: the increase in oxytocin production is not changed at all. In earlier studies [20,21] we observed the same phenomenon in connection with dopamine or serotonin regulation. 

Our present findings prove that the plasma oxytocin level is increased following ghrelin administration, and that the GHS-R1a ghrelin antagonist can block the enhancement of oxytocin secretion induced by i.c.v. or i.v. administered ghrelin. Both neurohypophyseal and plasma oxytocin secretion by ghrelin is a pivotal step in improving the understanding of oxytocin physiology and potential application. The importance of ghrelin/oxytocin neuropeptides at hypothalamus/pituitary sites promote new horizon for metabolic disorders. Although the basic mechanism of ghrelin-induced oxytocin secretion is verified, further studies are necessary to investigate the potential therapeutic option of oxytocin in special models. 

## 4. Materials and Methods

### 4.1. Experimental Protocol

The experiments were performed on 3- to 4-month-old male Wistar rats, ranging in weight from 180 to 250 g (bred in our animal house; breeding stock from the Laboratory Animal Insitute, Gödöllő, Hungary). The animal care and research protocols were in accordance with the guidelines of our university. In our experiment, all efforts were made to minimize the number of animals as well as the animal suffering.

Ghrelin was administered i.v. into the lateral tail vein or i.c.v. into the right lateral cerebral ventricle. For i.c.v. administration, the anesthetized rats were cannulated 7 days before the experiment. The animals were fixed in a stereotaxic instrument, a hole was drilled into the right parietal bone, and a cannula (Plastics One, Raonake, VA, USA) was inserted into the right lateral ventricle 0.7 mm anterior and 1.0 mm lateral to the bregma, according to the stereotaxic atlas. The length of the cannula was 4.0 mm, starting from the cranial bone. The cannula was fixed to the surface of the bone with rapidly hardening dental cement. 

For i.v. administration, the ghrelin dose was 1.0 or 10.0 nmol in 200 µL of saline. For the i.c.v. injection of ghrelin, the dose was 1.0, 10.0, or 100.0 pmol in 10 µL of saline in 1 min, via a Hamilton syringe. [d-Lys^3^]-GHRP-6 was administered i.c.v. in a dose of 10.0 pmol in 10 µL of saline in 1 min, 15 min before ghrelin administration. For i.v. administration of the ghrelin antagonist, the dose was 10.0 nmol in 200 µL of saline in 1 min, 15 min before ghrelin injection. In the control rats, 10 µL of physiological NaCl solution was administered i.c.v., and 200 µL of NaCl solution was administered i.v.

**Figure 5:**
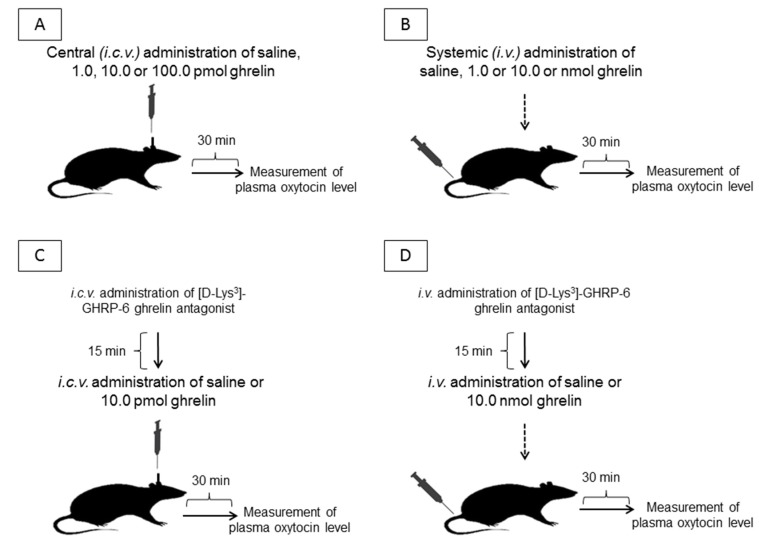
The experimental protocol of the study. i.c.v. = intracerebroventricular/central administration; i.v. = intravenous/systemic administration.

Thirty min following the administrations of ghrelin or ghrelin antagonist, the rats were decapitated and the blood was collected in cooled polystyrene tubes. The position of the i.c.v. cannula was checked by the injection of 1% methylene blue (Reanal, Budapest, Hungary) following coronal section of the brain. If the position of the cannula was not correct, these cases (6.5%) were excluded from the assessment of the results. The experimental protocol of the study is presented in Figure 5.

### 4.2. Plasma Oxytocin Determination

The oxytocin levels were measured by the RIA technique [5]. Synthetic oxytocin served as reference preparation for radiolabelling and specific oxytocin antibody was used (Bachem, CA, USA). Reverse-phase chromatography was used to purify the labelled hormone. The standard curves covered the range from 1.0 to 128 pg per assay tube. Oxytocin was extracted from supernatants with an Amprep C8 minicolumn (Code RPN 1902, Amersham, Buckinghamshire, UK), with a recovery of more than 95%. The dry residue was redissolved in 125 µL of assay buffer and 50-µL aliquots were used in duplicate for the RIA. The sensitivity of the assay for oxytocin was 1 pg/tube. Oxytocin values are reported in pg/mg protein. The intra- and interassay coefficients of variation proved to be 13.3% and 16.3%, respectively.

The following compounds were used; rat ghrelin (synthetized in the Department of Medical Chemistry, University of Szeged, Szeged, Hungary), [D-Lys^3^]-GHRP-6 (Tocris, Bristol, UK), oxytocin (oxytocin; Richter, Budapest, Hungary), and oxytocin antibody (Bachem, Torrance, CA, USA).

### 4.3. Protein Measurement

A modified Lowry method was used for the determination of total protein [20,23].

### 4.4. Statistical Analysis

The data are expressed as means ± S.E.M. of the results for the total number of rats per experimental group (GraphPad Prism 4). Statistical analysis was performed by using the Tukey–Kramer multiple comparison test. *p*-values less than 0.05 were considered significantly different.
